# Rapid inter-regional and cross-border dissemination of carbapenemase-producing *Klebsiella pneumoniae* ST6668, European Union, April 2022 to August 2026

**DOI:** 10.2807/1560-7917.ES.2026.31.43.2600861

**Published:** 2026-10-29

**Authors:** Giulia Errico, Marius Linkevicius, Alessandra Lo Sciuto, Cristiana Barbati, Sara Giancristofaro, Manuela Marra, Valentina Libri, Maria Del Grosso, Anette M Hammerum, Louise Roer, Rémy A Bonnin, Laurent Dortet, Felix Reichert, Lisa-Marie Höfken, Antoni PA Hendrickx, Dragos Florea, Daniela Talapan, Erik Alm, Olov Svartström, Priyanka Nannapaneni, Anke Kohlenberg, Monica Monaco, the Italian CRE25 survey working group for Klebsiella pneumoniae ST6668, Collaborators, Claudio Giacomazzi, Marisa Accogli, Maria Chironna, Floriana Gona, Maddalena Calvo, Stefania Stefani, Francesca Greco, Chiara Vismara, Teresa Lopizzo, Sofia Chiatamone Ranieri, Annarita Mazzariol, Luciano Castiello

**Affiliations:** 1 National Reference Laboratory for Antimicrobial Resistance, Department of Infectious Diseases, Istituto Superiore di Sanità, Rome, Italy; 2 European Centre for Disease Prevention and Control, Solna, Sweden; 3 Core Facilities Technical-Scientific Service, Istituto Superiore di Sanità, Rome, Italy; 4 National Reference Laboratory for Antimicrobial Resistance, Department of Bacteria, Parasites and Fungi, Statens Serum Institut, Copenhagen, Denmark; 5 European Union Reference Laboratory for Public Health on Antimicrobial Resistance in Bacteria (EURL-PH-AMR), Copenhagen, Denmark; 6 French National Reference Center for Antibiotic Resistance, Le Kremlin-Bicêtre, France; 7 Team 'Resist' UMR1184 'Immunology of Viral, Auto-Immune, Hematological and Bacterial Diseases (IMVA-HB)', INSERM, Université Paris-Saclay, CEA, LabEx LERMIT, Le Kremlin-Bicêtre Cedex, France; 8 Department of Bacteriology-Hygiene, Bicêtre Hospital, Assistance Publique-Hôpitaux de Paris, Le Kremlin-Bicêtre, France; 9 Department of Infectious Disease Epidemiology, Robert Koch Institute, Berlin, Germany; 10 German National Reference Centre for Multidrug-resistant Gram-negative Bacteria, Department of Medical Microbiology, Ruhr-University Bochum, Bochum, Germany; 11 Centre for Infectious Disease Control, National Institute for Public Health and the Environment (RIVM), Bilthoven, the Netherlands; 12 Carol Davila University of Medicine and Pharmacy, Bucharest, Romania; 13 National Institute for Infectious Disease “Prof. Dr. Matei Balş”, Bucharest, Romania; 14 The members of the Italian CRE25 survey working group for *Klebsiella pneumoniae* ST6668 are listed under Collaborators and at the end of the article.

**Keywords:** carbapenemase-producing *Klebsiella pneumoniae*, ST6668, European Union, genomic surveillance, cross-border spread

## Abstract

Preliminary analysis of genomic surveillance data from the European Antimicrobial Resistance Gene Surveillance Network detected the spread of the emerging carbapenemase-producing *Klebsiella pneumoniae* sequence type ST6668 in 19 NUTS-2 regions in Italy (8 regions) and five other European Union (EU) countries (11 regions) in the period April 2022 to August 2026. These findings demonstrate the vulnerability of EU healthcare facilities to the rapid spread of *K. pneumoniae* high-risk lineages from uncontrolled local outbreaks via national and cross-border patient mobility.

Carbapenem-resistant Enterobacterales (CRE) represent a major public health threat, with the dissemination of carbapenemase-producing *Klebsiella pneumoniae* high-risk clones contributing substantially to the spread of CRE across Europe [1]. Preliminary analysis of data from the third European genomic survey of carbapenem-resistant Enterobacterales 2025 (CRE25 survey) identified 19 closely related isolates of *K. pneumoniae* sequence type (ST) 6668, a single-locus variant of ST147, originating from Italy, Germany, France and Romania. *K. pneumoniae* ST6668 had not been detected in the previous two European genomic CRE surveys conducted in 2013–2014 [2] and 2019 [3]. On 18 August 2026, the European Centre for Disease Prevention and Control (ECDC) posted an alert on the emergence of ST6668 with a request for data on additional cases on the European surveillance portal for infectious diseases, EpiPulse, resulting in submission of 11 additional ST6668 isolates detected during routine genomic surveillance in European Union (EU) countries. Here, we report the epidemiological and genomic findings related to the dissemination of carbapenemase-producing *K. pneumoniae* ST6668 to enable rapid control measures.

## Data collection and analysis

Up to 3 July 2026, 31 of 37 invited countries from the European Union/European Economic Area (EU/EEA), Western Balkans and Türkiye had submitted epidemiological, microbiological and genomic data to ECDC for the CRE25 survey, covering isolates collected between 1 October and 31 December 2025, following a harmonised protocol with isolate collection intended to cover each nomenclature of territorial units for statistics level 2 (NUTS-2) region in participating countries [4]. Additional data was received after an EpiPulse alert on *K. pneumoniae* ST6668 from national routine surveillance with collection dates in the period April 2022 to August 2026. 

Bacterial identification and in vitro susceptibility testing were performed by the participating hospital laboratories using routine methods. Antimicrobial susceptibility testing (AST) results were interpreted in accordance with 2026 European Committee on Antimicrobial Susceptibility Testing (EUCAST) clinical breakpoints v16.1 [5]. Sequencing data for all isolates were processed at ECDC, as outlined in the CRE25 survey analysis plan [6]. 

On 28 August 2026, the National Center for Biotechnology Information (NCBI) was accessed to download publicly available genomes of *K. pneumoniae* ST6668 (n = 188; 187 from Italy and one from Switzerland) for genomic contextualisation: PRJEB64702, PRJNA1019273, PRJNA1238094, PRJNA1266098, PRJNA1345544, PRJNA1426723, PRJNA1449000, PRJNA1483592, PRJNA744003.

## Epidemiological and microbiological results

Fifteen *K. pneumoniae* ST6668 isolates were collected in Italy from eight of 19 hospital laboratories participating in the CRE25 survey enrolled within the framework of the national antibiotic-resistance surveillance network (AR-ISS) coordinated by Istituto Superiore di Sanità. The eight laboratories were geographically distributed across eight Italian NUTS-2 regions: Abruzzo, Basilicata, Calabria, Lombardy, Apulia, Sicily, Aosta Valley, and Veneto (Table, Figure 1). 

**Table:** Epidemiological characteristics associated with detected carbapenem-resistant *Klebsiella pneumoniae* ST6668 isolates, European Union, April 2022–August 2026 (n = 30)

Country	Isolate identifier	NUTS-2 region	Patient type	Hospital unit type	Prior hospitalisation	Prior residence in LTCF	Prior travel	Month and year of sampling	Specimen source	Clinical significance	Site of infection
Denmark	AMA007230	North Jutland	NA	NA	NA	NA	Yes, Italy	Jun 2025	Clinical	NA	NA
Denmark	AMA008372	Capital region	NA	NA	NA	NA	Yes, Italy	Aug 2026	Clinical	NA	NA
France	322H6	Rhone-Alpes	Inpatient	Geriatry	NA	NA	Yes, Italy	Apr 2022	Screening	Colonisation	N/A
France	490H7	Alsace	Inpatient	Nephrology	NA	NA	No	Apr 2024	Screening	Colonisation	N/A
France	610B7	Upper Normandy	Inpatient	Medical and rehabilitation care	NA	NA	No	Oct 2024	Screening	Colonisation	N/A
France	530A9	Alsace	Inpatient	Haematology	NA	NA	No	Mar 2025	Screening	Colonisation	N/A
France	578D5	Alsace	Inpatient	Nephrology	NA	NA	No	Sep 2025	Screening	Colonisation	N/A
France	601F10	Alsace	Inpatient	Infectious diseases department	NA	NA	No	Nov 2025	Screening	Colonisation	N/A
France	CRE25V_FRD2_KP09	Upper Normandy	Inpatient	Medical and rehabilitation care	NA	NA	Yes, Italy	Dec 2025	Screening	Colonisation	N/A
Germany	CRE25M_DE11_KP04	Stuttgart	Inpatient	NA	NA	NA	Yes, Tunisia	Oct 2025	Screening	NA	NA
Germany	CRE25M_DEA1_KP03	Düsseldorf	NA	NA	NA	NA	NA	Nov 2025	Clinical	NA	NA
Germany	NRZ-116303	Karlsruhe	NA	NA	Yes, Italy	NA	Yes, Italy	May 2026	Clinical	NA	NA
Italy	CRE25M_ITC4_KP01	Lombardy	Inpatient	Haematology/oncology	Yes, Italy	No	NA	Oct 2025	Clinical	Infection	Bloodstream
Italy	CRE25M_ITF1_KP03	Abruzzo	Inpatient	Intensive care unit	No	No	NA	Nov 2025	Clinical	Infection	Other
Italy	CRE25M_ITF1_KP07	Abruzzo	Inpatient	Emergency department	Yes, Italy	No	NA	Nov 2025	Clinical	Infection	Bloodstream
Italy	CRE25M_ITC2_KP01	Aosta Valley	Outpatient	Emergency department	Yes, Italy	Yes, Italy	NA	Nov 2025	Clinical	Infection	Bloodstream
Italy	CRE25M_ITC2_KP03	Aosta Valley	Outpatient	Other	Yes, Italy	Yes, Italy	NA	Nov 2025	Clinical	Undetermined/unknown	N/A
Italy	CRE25M_ITF4_KP03	Apulia	Inpatient	Inpatient ward	NA	No	NA	Nov 2025	Clinical	Infection	Bloodstream
Italy	CRE25M_ITF4_KP05	Apulia	Inpatient	Internal medicine	NA	No	NA	Nov 2025	Clinical	Infection	Bloodstream
Italy	CRE25M_ITF6_KP02	Calabria	Inpatient	Other	Yes, Italy	NA	NA	Nov 2025	NA	Undetermined/unknown	N/A
Italy	CRE25M_ITF6_KP03	Calabria	Inpatient	Urology ward	Yes, Italy	NA	NA	Nov 2025	NA	Infection	Bloodstream
Italy	CRE25M_ITC4_KP07	Lombardy	Inpatient	Urology ward	NA	NA	NA	Nov 2025	Clinical	Infection	Urinary tract
Italy	CRE25M_ITG1_KP09	Sicily	Inpatient	Internal medicine	NA	NA	NA	Nov 2025	Clinical	Infection	Bloodstream
Italy	CRE25M_ITH3_KP02	Veneto	Inpatient	Surgery	Yes, Italy	NA	NA	Nov 2025	Screening	Colonisation	Other
Italy	CRE25M_ITF5_KP08	Basilicata	Inpatient	Surgery	NA	NA	NA	Dec 2025	Clinical	NA	N/A
Italy	CRE25M_ITC4_KP10	Lombardy	Inpatient	Urology ward	NA	NA	NA	Dec 2025	Clinical	Infection	Urinary tract
Italy	CRE25M_ITH3_KP09	Veneto	Inpatient	Surgery	NA	No	NA	Dec 2025	Clinical	Infection	Urinary tract
Netherlands	RIVM_C095401	North Holland	NA	NA	Yes, Italy	NA	Yes, Italy	Jun 2025	Screening	NA	NA
Netherlands	RIVM_C098775	Gelderland	NA	NA	Yes, Italy	NA	Yes, Italy	Sep 2025	Screening	NA	NA
Romania	CRE25M_RO32_KP07	Bucharest-Ilfov	Inpatient	Infectious disease ward	Yes, Romania	No	No	Oct 2025	Clinical	Infection	Urinary tract

LTCF: long-term care facility; NA: data not available; NUTS-2: nomenclature of territorial units for statistics level 2; N/A: not applicable.

**Figure 1:**
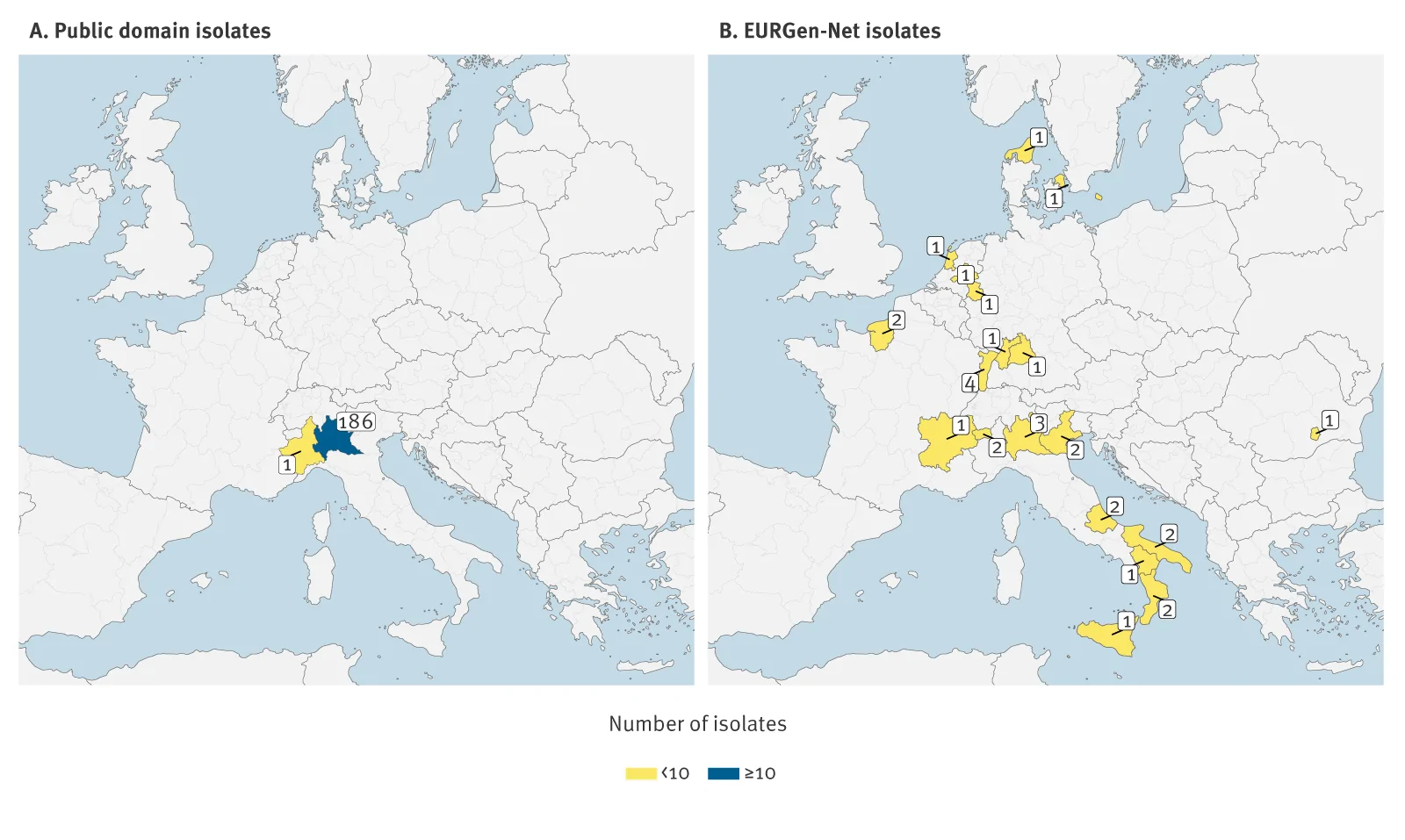
Geographic distribution of *Klebsiella pneumoniae* ST6668 isolates (A) available in the public domain (n = 187^a^) from Italy, 2020–2025, and (B) from EURGen-Net genomic surveillance (n = 30), European Union, 2022–2026

EURGen-Net: European Antimicrobial Resistance Genes Surveillance Network; NUTS-2: nomenclature of territorial units for statistics level 2.

^a^ One isolate from Switzerland did not have specified statistical region equivalent for NUTS-2 region and therefore was not marked on the map.

Fifteen isolates originated from other EU countries: France (n = 7 from Alsace, Upper Normandy and Rhone-Alpes regions), Germany (n = 3 from Düsseldorf, Karlsruhe and Stuttgart regions), Denmark (n = 2 from North Jutland and the Capital regions), the Netherlands (n = 2 from Gelderland and North Holland regions) and Romania (n = 1 from Bucharest-Ilfov region) (Figure 1). Epidemiological characteristics of the cases are outlined in the Table. Included isolates were from 16 male and 14 female patients in the following age groups: 19–64 years (n = 7), ≥ 65 years (n = 22). For one isolate, age group was not available.

Clinical significance was determined for 20 of the 30 cases. Of these, 12 were associated with infection (n = 11 from Italy and n = 1 from Romania) while eight isolates (n = 7 from France and n = 1 from Italy) were associated with colonisation. The most common types of infections were bloodstream infections (n = 7) and urinary tract infections (n = 4). Information on prior hospital admission, residence in long-term care facilities and travel within the previous 6 months was incomplete. Nevertheless, seven of 15 cases reported from countries other than Italy had a documented travel history to or prior hospitalisation in Italy and one a travel history to Tunisia (Table).

All isolates with available AST data (n = 21) were resistant to at least one carbapenem. Of the 17 isolates tested against multiple antimicrobial categories, all were multidrug-resistant. Resistance to piperacillin/tazobactam, ceftazidime and ciprofloxacin was detected in 17 and to tobramycin in 16 tested isolates. Resistance to ceftazidime/avibactam was observed in 13 of 17 isolates, compared with 12 of 16 tested isolates for both meropenem/vaborbactam and imipenem/relebactam. Resistance to gentamicin was rare (2/17), while no resistance to colistin was detected (0/16).

## Genomic characteristics and phylogeny

All 30 analysed *K. pneumoniae* ST6668 isolates carried at least one carbapenemase gene. The most prevalent carbapenemase was *bla*_NDM-1_, detected in 25 isolates, followed by *bla*_KPC-3_ in four isolates. One isolate co-harboured *bla*_KPC-2_ and *bla*_NDM-1_. Additional plasmid-borne resistance genes, such as extended-spectrum beta-lactamase genes, were found in 21 isolates, all represented by the *bla*_CTX-M-15_ gene (a list of genes for each isolate is provided in Supplementary Table S1). All isolates carried mutations in outer membrane porins that contribute to carbapenem resistance (truncated OmpK35 and Gly134Asp135 duplication in OmpK36, n = 29; or only truncated OmpK35, n = 1). All isolates exhibited a Kleborate [7] virulence score of 1 based on the presence of yersiniabactin lineage *ybt9*.

Single-linkage clustering at 10 allelic differences including also the contextual publicly available data showed that the 30 *K. pneumoniae* ST6668 isolates were part of two genetic clusters (Figure 2) together with isolates from the previously described outbreaks of *K. pneumoniae* ST6668 in northern Italy [8-10] (single-linkage dendrogram depicting clusters can be found in Supplementary Figure S1). The larger cluster (Cluster 1) included 13 isolates from eight NUTS-2 regions in Italy (Abruzzo, Basilicata, Calabria, Lombardy, Apulia, Sicily, Aosta Valley, and Veneto) as well as 13 isolates from the other countries including France (n = 6), Germany (n = 3), the Netherlands (n = 2), Denmark (n = 1) and Romania (n = 1). The remaining four isolates (n = 2 from Italy, n = 1 from Denmark and n = 1 from France) were part of a smaller cluster (Cluster 2). 

**Figure 2:**
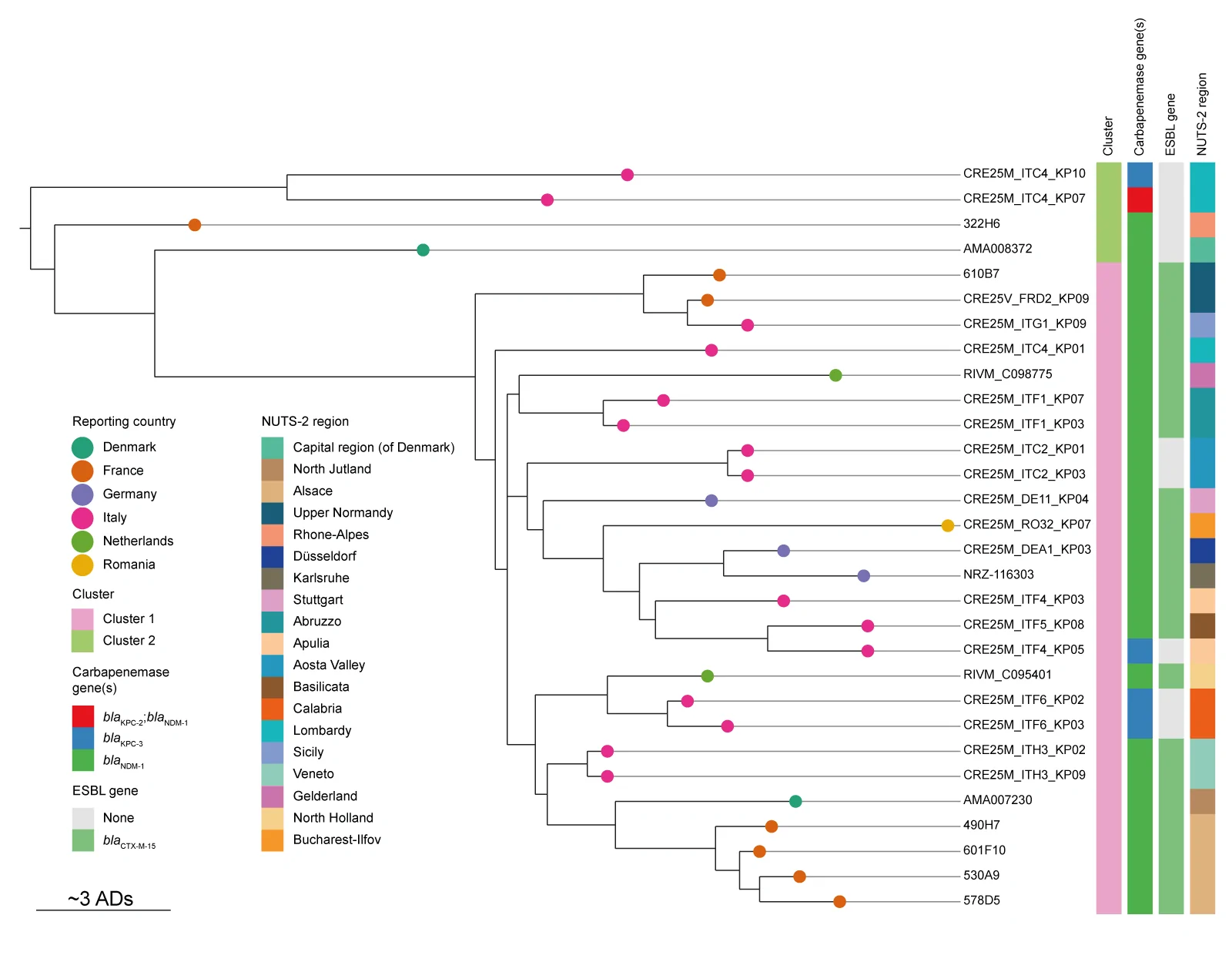
Phylogenetic tree of *Klebsiella pneumoniae* ST6668 EURGen-Net genomic surveillance isolates, European Union, April 2022–August 2026 (n = 30)

EURGen-Net: European Antimicrobial Resistance Genes Surveillance Network; NUTS-2: nomenclature of territorial units for statistics level 2.

Clustering of the 30 *Klebsiella* *pneumoniae* ST6668 isolates in this investigation was performed using a single-linkage algorithm with a 10 allelic differences (AD) cut-off including also the contextual publicly available data and this resulted in two single-linkage clusters (see the single-linkage dendrogram in Supplementary Figure S1). Pairwise distances within a single-linkage cluster can be larger than the cut-off used. In the displayed neighbour joining tree, the root-to-tip distance was 23 ADs, with pairwise distances varying between 1–19 ADs and 12–25 ADs in Cluster 1 and Cluster 2, respectively. Rooting of the tree was based on ST147 outgroup.

The 215 *K. pneumoniae* ST6668 isolates with available sampling dates (n = 185 from the public domain and n = 30 from this study) covered a period from October 2020 to August 2026 (Figure 3).

**Figure 3:**
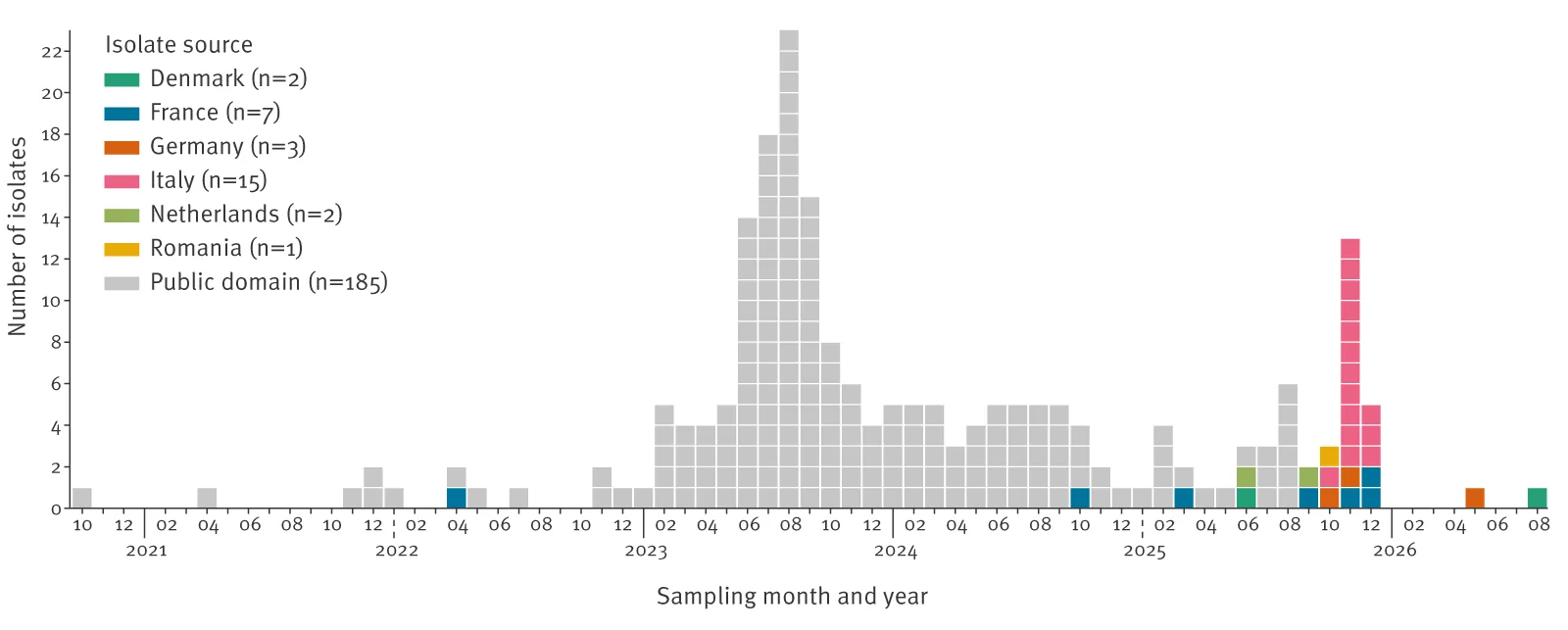
Time distribution of *Klebsiella pneumoniae* ST6668 EURGen-Net genomic surveillance isolates (n = 30) compared with publicly available isolates (n = 185^a^), European Union, October 2020–August 2026

EURGen-Net: European Antimicrobial Resistance Genes Surveillance Network.

^a^ Three isolates from the public domain did not contain sampling dates and were excluded from the plot: one isolate from Switzerland and two isolates from Italy were sampled in 2020 and 2024, respectively. All the other public domain isolates included in the plot (n = 185) originated from Italy.

The time distribution illustrated above should not be interpreted as an epidemic curve, as year-to-year variation is affected by detection and reporting biases and may not reflect true temporal trends in incidence.

## Discussion

Our analysis documents the spread of *K. pneumoniae* ST6668 between regions in Italy and cross-border to five EU countries. The novel NDM-producing *K. pneumoniae* ST6668 clone, a single-locus variant of ST147, was identified during an outbreak involving two healthcare facilities in northern Italy in 2023 [8]. At the time, its circulation appeared to be confined to a local healthcare network [8-11]. In 2025, another *K. pneumoniae* ST6668 isolate was also reported in northern Italy [12], indicating that the clone may have persisted beyond the initial outbreak setting. Data from the CRE25 survey revealed the presence of *K. pneumoniae* ST6668 in multiple regions across both northern and southern Italy, indicating a substantially wider geographical distribution than previously recognised. A limitation of this manuscript intended as a rapid alert is that detailed epidemiological metadata and microbiological data (AST) results were incomplete at the time of publication.

Patient mobility between regions may have contributed to the dissemination within Italy. Patients from southern regions frequently access highly specialised services in northern referral hospitals and subsequently return to their region of residence for follow-up care or further hospitalisation, reflecting established patterns of interregional healthcare mobility within the Italian national healthcare service [13,14]. Such movements may facilitate not only the introduction of multidrug-resistant lineages into geographically distant healthcare settings but also their subsequent dissemination within local healthcare networks, as patient transfers are a well-recognised driver of regional and interregional spread of *K. pneumoniae* high-risk clones [3]. The presence of *K. pneumoniae* ST6668 in multiple southern Italian regions may reflect a combination of interregional patient mobility and secondary transmission following local introduction. Detection of ST6668 in five additional countries, given its recent emergence, underscores the relevance of cross-border transfers within the EU.

Our findings highlight the need for strengthened infection prevention and control (IPC) strategies at both regional and national levels, in line with the recommendations outlined in the Italian Ministry of Health circular on the surveillance and control of CRE [15]. In the context of interregional healthcare mobility and cross-border patient movement, strict implementation of these recommendations is essential to limit further dissemination of emerging clones such as ST6668. Particular emphasis should be placed on active screening of high-risk patients, contact precautions, hand hygiene, environmental decontamination and communication of carrier status during patient transfers, especially within highly connected healthcare networks.

The detection of the emerging ST6668 clone also highlights the importance of integrated genomic surveillance at the national and EU level and of the EpiPulse platform for rapid alerts. Coordinated genomic monitoring capable of linking cases across healthcare networks could facilitate earlier recognition of emerging outbreak-related strains, improve understanding of transmission pathways, and provide actionable information to support targeted containment measures. 

## Conclusion

The emergence of ST6668 highlights how healthcare connectivity may contribute to the rapid dissemination of novel carbapenem-resistant *K. pneumoniae* clones within Europe. Together, strengthened IPC practices including screening of patients with prior healthcare contact and nationwide genomic surveillance will be critical to limiting the further spread of *K. pneumoniae* ST6668 and other emerging carbapenem-resistant *K. pneumoniae* lineages in Italy and beyond.

## Data Availability

Genomic data collected for this report are accessible under PRJEB75178, PRJNA1530922, PRJEB127206.

## The Italian CRE25 survey working group for *Klebsiella pneumoniae* ST6668

Aosta, CLAUDIO GIACOMAZZI (Ospedale Regionale “U. Parini”); Bari, MARISA ACCOGLI, MARIA CHIRONNA (AOUC Policlinico di Bari); Catania, FLORIANA GONA, MADDALENA CALVO, STEFANIA STEFANI (Azienda Ospedaliero Universitaria Policlinico “G. Rodolico - San Marco”); Cosenza, FRANCESCA GRECO (Azienda Ospedaliera Cosenza- Presidio Ospedaliero Annunziata); Milano, CHIARA VISMARA (ASST Grande Ospedale Metropolitano Niguarda); Potenza, TERESA LOPIZZO (Azienda Ospedaliera Regionale San Carlo Potenza); Teramo, SOFIA CHIATAMONE RANIERI (Asl Teramo); Verona, ANNARITA MAZZARIOL (Azienda Ospedaliera Universitaria Integrata di Verona); Roma, LUCIANO CASTIELLO (Istituto Superiore di Sanità, Core Facilities Technical-Scientific Service).

## Supplementary Data

620000 Supplementary Figure

## Supplementary Data

39000 Supplementary Table
