# Supplementary material for: Rapid inter-regional and cross-border dissemination of carbapenemase-producing Klebsiella pneumoniae ST6668, European Union, April 2022 to August 2026
Source: Euro Surveill. 2026 Oct 29;31(43):2600861. doi: 10.2807/1560-7917.ES.2026.31.43.2600861 (PMC13722929; doi:10.2807/1560-7917.ES.2026.31.43.2600861)

Rapid inter-regional and cross-border dissemination of carbapenemase-producing *Klebsiella pneumoniae* ST6668, European Union, April 2022 to August 2026

Supplementary materials

This supplementary material is hosted by Eurosurveillance as supporting information alongside the article "Rapid inter-regional and cross-border dissemination of carbapenemase-producing *Klebsiella pneumoniae* ST6668, European Union, April 2022 to August 2026", on behalf of the authors, who remain responsible for the accuracy and appropriateness of the content. The same standards for ethics, copyright, attributions and permissions as for the article apply. Supplements are not edited by Eurosurveillance and the journal is not responsible for the maintenance of any links or email addresses provided therein.

SUPPLEMENTARY FIGURE S1. Single-linkage tree of *Klebsiella pneumoniae* ST6668 EURGen-Net genomic surveillance isolates (n = 30) in the context of publicly available isolates (n = 188), European Union and Switzerland, 2020–2026

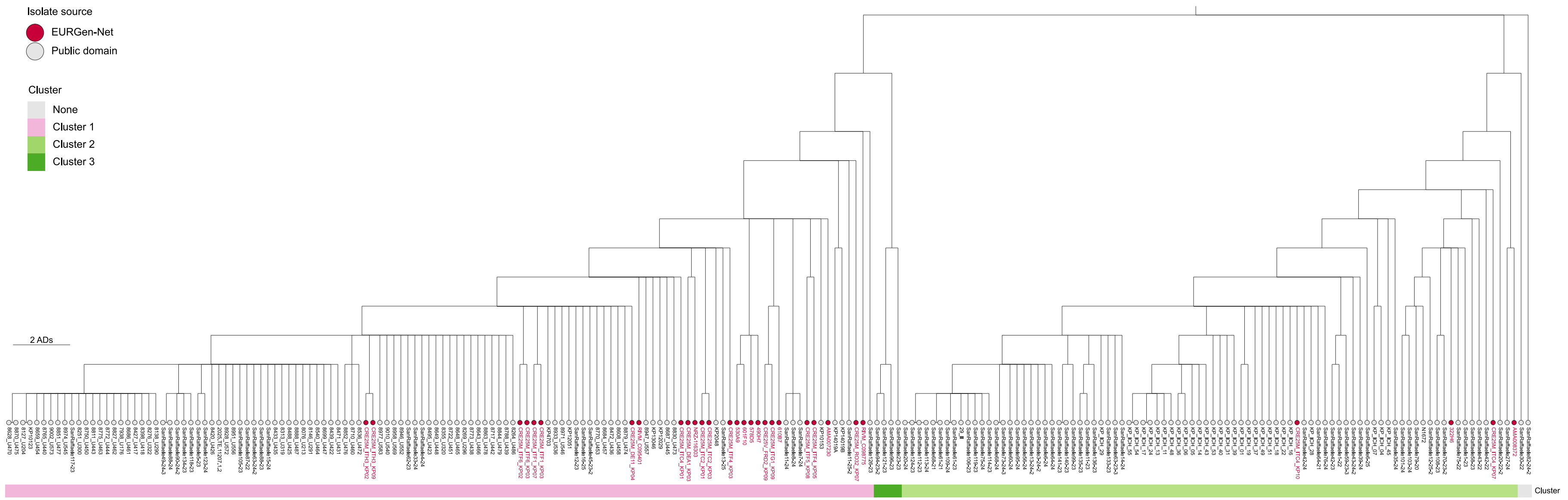

Supplement: Supplementary file 1 [file 26-00861_ERRICO_Supplementary_Figure.pdf]
